# Rv2190c, an NlpC/P60 Family Protein, Is Required for Full Virulence of *Mycobacterium tuberculosis*

**DOI:** 10.1371/journal.pone.0043429

**Published:** 2012-08-31

**Authors:** Geetha Parthasarathy, Shichun Lun, Haidan Guo, Nicole C. Ammerman, Deborah E. Geiman, William R. Bishai

**Affiliations:** 1 Center for Tuberculosis Research, Johns Hopkins University School of Medicine, Baltimore, Maryland, United States of America; 2 Howard Hughes Medical Institute, Chevy Chase, Maryland, United States of America; University of Padova, Italy

## Abstract

*Mycobacterium tuberculosis*, the etiologic agent of tuberculosis (TB) possesses at least five genes predicted to encode proteins with NlpC/P60 hydrolase domains, including the relatively uncharacterized *Rv2190c*. As NlpC/P60 domain-containing proteins are associated with diverse roles in bacterial physiology, our objective was to characterize *Rv2190c* in *M. tuberculosis* growth and virulence. Our data indicate that lack of *Rv2190c* is associated with impaired growth, both *in vitro* and during an *in vivo* mouse model of TB. These growth defects are associated with altered colony morphology and phthiocerol dimycocerosate levels, indicating that Rv2190c is involved in cell wall maintenance and composition. In addition, we have demonstrated that *Rv2190c* is expressed during active growth phase and that its protein product is immunogenic during infection. Our findings have significant implications, both for better understanding the role of Rv2190c in *M. tuberculosis* biology and also for translational developments.

## Introduction

Approximately one-third of the world's population is estimated to be infected with *Mycobacterium tuberculosis*, the causative agent of tuberculosis (TB) [1]. Infection, disease and death due to this pathogen are devastating, with the highest burdens spread across Africa and Asia [2]. The lack of accurate point-of-care diagnostics and an efficacious vaccine, combined with the ever-increasing development of drug-resistant strains, subverts efforts to control TB around the globe. Thus, there is a desperate need for new drug targets, as well as for *M. tuberculosis*-specific markers for diagnostic and vaccine development.

Recent studies with other bacteria have indicated that proteins of the NlpC/P60 superfamily are important in bacterial physiology. The NlpC/P60 domain (named after New lipoprotein C from *Escherichia coli* and a 60 kDa extracellular protein of *Listeria monocytogenes*) provides hydrolase activity, and proteins with this domain include endopeptidases, amidases and acyltransferases involved in growth, development, cell division, cell wall maturation, and also virulence [3]–[6]. Based on genomic information, *M. tuberculosis* is predicted to encode at least five proteins with NlpC/P60 domains: Rv0024, Rv1477, Rv1478, Rv1566c and Rv2190c [7]. Their amino acid sequences (with the exception of Rv1566c) indicate a hydrolase domain containing the signature motifs “DCSG” and “GD” in the C-terminus with a conserved histidine residue further downstream. This family of proteins has not been well studied in *M. tuberculosis* and thus represents new possibilities for development of drug targets, diagnostics and vaccines.

In *M. marinum*, a mycobacterial pathogen of fish and amphibians, the orthologues of *Rv1477* and *Rv1478*, termed *iipA* and *iipB*, respectively, were both shown to be required for the invasion of macrophages [4]. In addition, *iipA* was shown to be essential for virulence, cell separation, resistance to antibiotics and lysozyme, and persistence in the zebrafish model, while *iipB* was required for cell separation and cording, in addition to macrophage invasion. In *M. tuberculosis*, limited work has been done with this family of proteins. Rv1477 (RipA) has been shown to be a Sec-dependent secreted protein with hydrolase activity and the ability to degrade peptidoglycan [8]. The C-terminus of this endopeptidase has been shown to interact with the resuscitation-promoting factors B and E (RpfB and RpfE, respectively), two lytic transglycosylases also with peptidoglycan hydrolase activity that have been implicated in *M. tuberculosis* revival from dormancy [9], [10]. Both RpfB and RipA have been shown to localize to the septa of dividing bacteria, thus suggesting a role in cell wall hydrolysis during cell division [10], [11]. In subsequent studies it was shown that RipA also interacts with a cell wall synthesizing protein, penicillin binding protein 1 (PBP1), through its C-terminus, indicating that cell wall synthesis and hydrolysis are likely coordinated through RipA [12]. Thus, the limited data available with regard to the role of NlpC/P60 proteins in *M. tuberculosis* are restricted to Rv1477, with little or no information available for the other genes or their products. Målen and colleagues have also identified Rv2190c in a mixture of 257 proteins in *M. tuberculosis* culture filtrate, suggesting that Rv2190c is expressed and secreted under *in vitro* conditions [13].

The *Rv2190c* gene is described as encoding a “hypothetical protein” containing an NlpC/P60 domain. Based on this description and the finding that this protein was expressed and secreted during *M. tuberculosis* growth in broth culture, we hypothesized that Rv2190c may be involved in *M. tuberculosis* growth and cell wall composition. In this study, we characterize the role of Rv2190c using both *in vitro* and *in vivo* experiments, and our results indicate that this NlpC/P60 family protein of *M. tuberculosis* has potential for the development of translational tools for TB.

## Materials and Methods

### Strains and growth conditions

*Mycobacterium tuberculosis* strain CDC1551 (wild-type) and an isogenic *Rv2190c* transposon mutant strain (JHU2190c-1147) were routinely grown in Middlebrook 7H9 medium supplemented with 0.5% glycerol, 0.05% Tween 80, 10% ovalbumin-dextrose-saline and 100 µg/ml cycloheximide, at 37°C with agitation. The mutant strain was constructed by phage-mediated transposon insertion mutagenesis; a single colony was isolated, genotyped by transposon-chromosome junctional PCR, and archived at −80°C as described [14]. The transposon, containing a kanamycin resistance gene, was inserted at base 1147 of *Rv2190c* just downstream of the predicted NlpC/P60 domain, corresponding to the C-terminus of the encoded protein product. Growth assays were conducted by dilution of exponentially growing stock cultures of bacteria to an initial OD_600_ of 0.05, and growth was measured by absorbance every two days.

### Assessment of *Rv2190c* mRNA expression during wild-type growth

To determine the expression of *Rv2190c* during the growth of the wild-type strain, *M. tuberculosis* CDC1551 RNA was extracted using Trizol reagent (Invitrogen) at various OD_600_, and *Rv2190c* mRNA was analyzed by real-time, quantitative RT-PCR. To enhance the concentration of total RNA, the cultures were subjected to mechanical shearing using silica and a bead-beater following the addition of Trizol reagent, centrifuged at 14,000 rpm 3 minutes at 4°C, and RNA was extracted according the manufacturer's instructions. Following the extraction, 1 µg of RNA was subjected to DNase I (Invitrogen) treatment and reverse-transcribed using iScript cDNA synthesis kit (BioRad). *Rv2190c* mRNA was analyzed by SYBR green real-time RT-PCR (BioRad) using forward primer 5′ - GCT GGA TCT CAA CGA AAA GC - 3′ and reverse primer 5′ - GGG TAC GAC CAC CCA TGT AG - 3′. *Rv2190c* expression was normalized to *sigA*, as previously described [15].

### Stress assay

The role of *Rv2190c* in stress survival of *M. tuberculosis* was evaluated by analysis of its mRNA expression in various stress conditions, according to Geiman *et al.* [16]. Briefly, wild-type *M. tuberculosis* CDC1551 grown to an OD_600_ of 0.4 were split into 50 ml aliquots, equilibrated for 2 hr at 37°C, and exposed to various stresses for 1.5 hr. Culture without any additional stress was used as a negative control. RNA was extracted, and real-time, quantitative RT-PCR was conducted as above-described. The CT values in each condition were normalized to internal *sigA*, and fold change over negative control (culture with no stress) was determined.

### Transmission electron microscopy (TEM)

To determine the effect of mutation on cell separation, the wild-type *M. tuberculosis* CDC1551 and the isogenic *Rv2190c* mutant strains were processed for TEM according to Pilgrim *et al.* [17] at the JHU microscopy core facility, and micrographs were assembled using Adobe Photoshop CS3.

### Lysozyme assay

To determine the effect of lysozyme on cell wall integrity, the wild-type CDC1551 strain, the *Rv2190c* mutant and complement strains of similar OD_600_ were divided, and half of the culture was incubated with 1 mg/ml of lysozyme for 24 hrs, and viability was assessed by cell count. The effect of lysozyme on cell survival was calculated as a percent of the viability without lysozyme treatment.

### Construction of a complemented mutant

The complement of *Rv2190c* was constructed using the Gateway BP cloning system (Invitrogen) according to the manufacturer's protocols. Briefly, the *Rv2190c* coding region along with 500 bp upstream was amplified by PCR using primers 5′ - GGG GAC AAG TTT GTA CAA AAA AGC AGG CTT CGA TGT CGG TGC TCG CGA TTA - 3′ and 5′ - GGG GAC CAC TTT GTA CAA GAA AGC TGG GTA TCA GTA ACG GCG GGC GTC GT - 3′ and cloned into pDONR/Zeo^R^ vector by the BP cloning reaction according to the manufacturer's instructions. The recombinant clones were verified by PCR and sequencing, and subsequently cloned into the destination vector pGS202/Hyg^R^ [18]. Resulting plasmids were purified and transformed into the electro-competent *Rv2190c* mutant, and positive clones selected on Kan^+^/Hyg^+^ 7H10 plates.

### Construction of an *Rv2190c* expression vector for protein purification

The sequence encoding the C-terminus of Rv2190c, containing the Nlpc/P60 domain, was cloned into N-terminal His-tag vector pET15b using primers 5′ - GAC CCA TAT GGT GGC GCC GCC GCC TGG TGG - 3′ and 5′ - CGC ACT CGA GTC AGT AAC GGC GGG CGT CGT - 3′. The vector sequence was verified by PCR, restriction enzyme digest and sequencing. Protein was expressed in *E. coli* BL21 (DE3) using MagicMedia™ (Invitrogen) and purified by nickel-NTA sepharose column by standard protocols.

### Phthiocerol dimycocerosate (PDIM) assay

Analysis of PDIM was conducted by one-dimensional thin layer chromatography of the mycobacterial strains. Briefly, cultures grown to mid-log phase (OD_600_ of 1.0), were harvested and autoclaved. Dry weights were measured to ensure similarity. Total lipids were extracted by the addition of 2∶1 vol/vol of chloroform/methanol, vortexed vigorously for 5 minutes and centrifuged for phase separation. Equal volumes of the lower organic phase were spotted on a TLC plate (silica gel), and separated either by hexane∶diethyl ether∶acetic acid (80∶20∶1vol/vol/vol) or petroleum ether∶diethyl ether (90∶10 vol/vol) and visualized with phosphomolybdic acid and heat.

### Dot-Blot assay

Immunogenic properties of Rv2190c were determined by dot-blot ELISA using purified protein and standard protocols. Briefly, 2 ng of N-terminal truncated Rv2190c or an equivalent bovine serum albumin (BSA) control were spotted on nitrocellulose membrane and equilibrated in Tris-buffered saline containing 0.05% Tween 20 (TBST). The membrane was blocked with 3% BSA in TBST for 1 hr at room temperature, washed and probed with various test and control sera (1∶1000, 1 hr, room termperature). An appropriate secondary antibody (anti-rabbit or anti-human) conjugated with horseradish peroxidase (1∶1000, 1 hr, room temperature) was used to capture the primary antibody, and the spots were developed with a chemiluminescent substrate and radiographed. The human sera were obtained from volunteers who were not vaccinated with *M. bovis* BCG and were known to be either TB skin test (purified protein derivative [PPD]) positive or negative. These serum samples were part of our laboratory collection. For the samples used in this study, we obtained oral and written consent under an approved protocol for utilization of human sera in *M. tuberculosis* proteomics-related studies. This protocol was approved by the Johns Hopkins Institutional Review Board. All sera were tested anonymously. The rabbit sera were also part of our laboratory stocks, obtained from the *M. bovis*-infected New Zealand White rabbits used in our previous work, reported by Nedeltchev *et al.* [19]. All rabbit work was also performed in accordance with protocols approved by the Johns Hopkins University Animal Care and Use Committee.

### Time-to-death studies

Groups of 16 six-week-old female BALB/c mice (Charles River) were aerosol-infected with the *Mycobacterium tuberculosis Rv2190c* mutant, the complemented strain and parental wild-type CDC1551 using the Middlebrook inhalation exposure system (Glas-Col) with log-phase broth cultures. Day 1 implantation was determined by sacrificing 3 mice for each group, and colony forming units (CFUs) in the lungs were enumerated. Death was recorded for each group and logrank comparison was conducted.

### *In vivo* studies: growth and pathology

To determine the role of *Rv2190c* during *in vivo* growth, the wild-type, mutant and complemented strains were tested in a mouse model. For lung pathology study, groups of 24 six-week-old female BALB/c mice were aerosol infected with a low dose of the *Mycobacterium tuberculosis Rv2190c* mutant, the complemented strain and parental wild-type CDC1551 using the methods above-described. At day 1, 7, 14, 28, 56, 84 and 112 after infection, four mice from each group were sacrificed, and the lungs and spleens were collected. The lung and spleen gross pathology were recorded, and the lung weight, spleen weight and body weight were also recorded. CFUs in the lungs and spleens of all time points were enumerated by homogenizing the tissues and plating out the dilutions onto 7H11 selective agar plates. Small portions of the lungs were collected and fixed with 10% formalin. Lung tissue sectioning and staining were done for histopathology analysis. All animal procedures were approved by the Johns Hopkins University Animal Care and Use Committee (protocol M011M120: Virulence testing by aerosol infection).

### Statistics

For the *in vitro* experiments, the paired T-test was conducted to determine statistical significance between two groups, when required.

## Results

### Interruption of *Rv2190c* influences growth and morphology phenotypes

**Figure 1:**
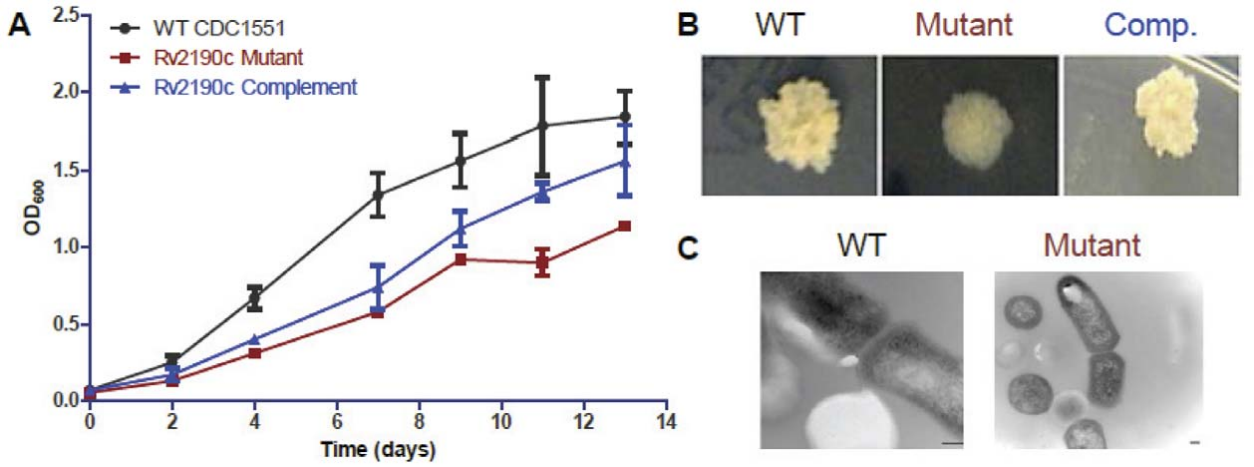
*Rv2190c* influences *M. tuberculosis* growth and colony morphology, but not cell septation. **A.** Growth curves in 7H9 broth for wild-type CDC1551 (WT), *Rv2190c* mutant and complement strains of *M. tuberculosis*. **B.** *M. tuberculosis* colony morphology on 7H11 agar plates for the wild-type, *Rv2190c* mutant and complement strains. **C.** Transmission electron micrographs of the wild-type and mutant *M. tuberculosis*. Scale bar = 100 nm.

To analyze the role of *M. tuberculosis Rv2190c*, we utilized a transposon insertion mutant that had been previously generated in our laboratory. This mutant strain contains the *Himar1* transposon with a kanamycin resistance marker inserted at position 1147 in *Rv2190c*, placing the insertion just after the predicted NlpC/P60 domain at the C-terminus of the cognate protein. Compared to the wild-type parent strain of *M tuberculosis.*, the *Rv2190c* mutant was attenuated for growth *in vitro* (Figure 1A). By day 13, the difference in growth rates between the two strains was statistically significant (p<0.05). The *Rv2190c* complemented strain exhibited intermediate growth in broth culture, possibly due to the presence of the truncated mutant protein competing with the full-length complement protein. However, gross morphology of the complemented strain was similar to wild-type, while the *Rv2190c* mutant displayed an altered morphology with smoother colony edges and surface compared to wild-type or complement strains (Figure 1B). This phenotype was observed on both 7H10 and 7H11 selective agar plates, indicating that the morphology change was not due to nutritional alterations (data not shown). As proteins in the NlpC/P60 family have been associated with septation defects in other bacterial species [4], [17], [20], we used TEM to characterize the septation phenotype of the *Rv2190c* mutant. As shown in Figure 1C, the *Rv2190c* mutant did not exhibit changes in septation compared to wild-type *M. tuberculosis* These initial data suggested that *Rv2190c* influences the *M. tuberculosis* extracellular interface in a manner that does not affect septation but does attenuate growth *in vitro*.

### *Rv2190c* expression peaks during log phase growth and is involved in the response to cell wall disruption

**Figure 2:**
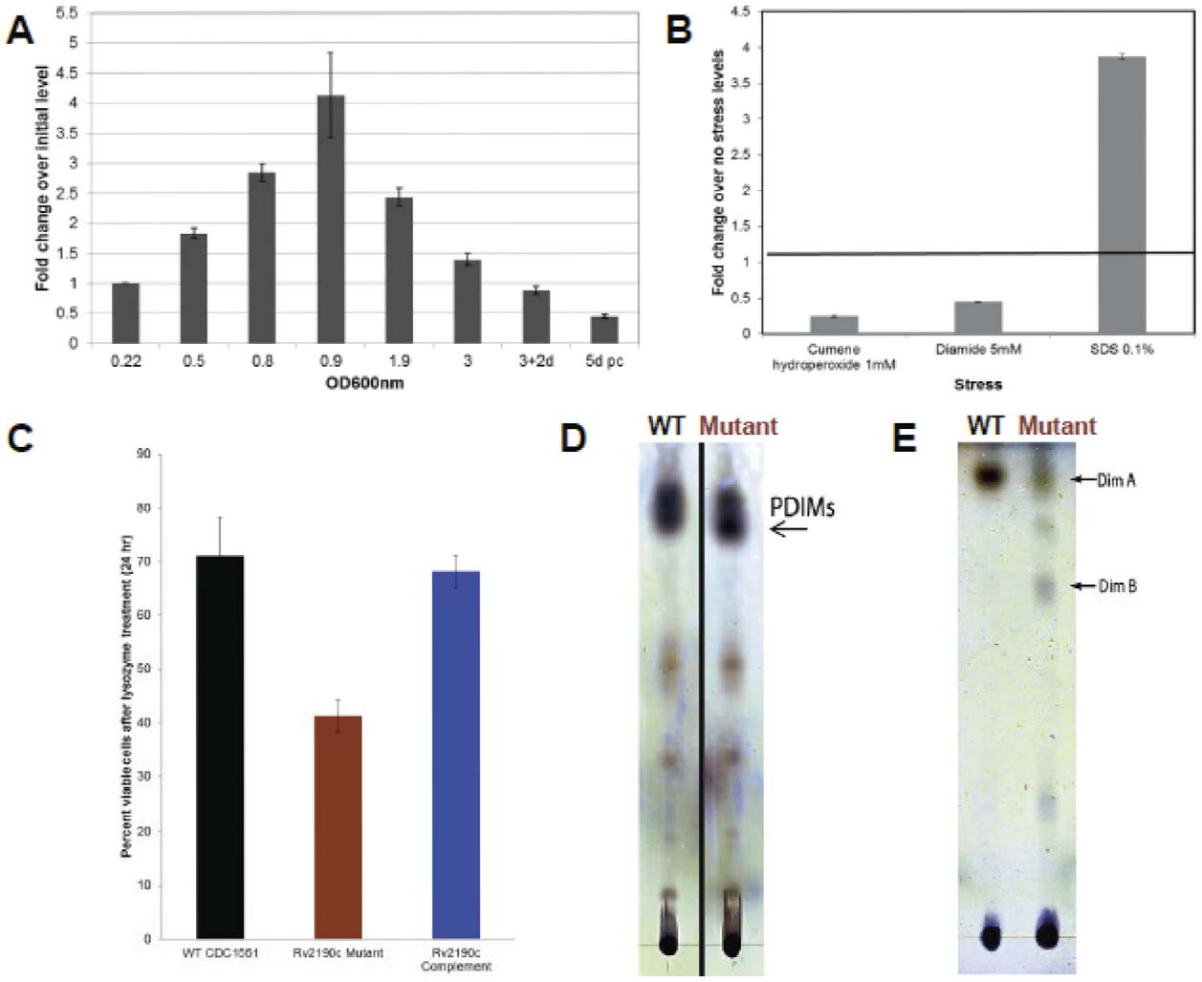
*Rv2190c* expression peaks at log phase and is involved in the response to cell wall disruption. **A.** Fold change of *Rv2190c* expression (relative to level at the start of the growth curve) over increasing cell density in 7H9 broth in wild-type *M. tuberculosis* CDC1551, normalized to *sigA* expression. pc: post-clumping. **B.** Fold-change of *Rv2190c* expression after exposure to oxidative (cumene), disulfide (diamide) and detergent (SDS) stress conditions, relative to expression levels prior to addition of stressors and normalized to *sigA*. **C.** Survival of WT, the *Rv2190c* mutant and complement *M. tuberculosis* strains following 24 hours of lysozyme exposure. **D.** PDIMs for *M. tuberculosis* WT and Rv2190c mutant strains analyzed by thin layer chromatography using hexane∶diethyl ether∶acetic acid solvent. **E.** PDIMs analyzed using petroleum ether∶diethyl ether solvent.

To further characterize the role of *Rv2190c* in *M. tuberculosis* biology, we measured *Rv2190c* gene expression during different phases of *M. tuberculosis in vitro* growth. *Rv2190c* transcript levels increased (relative to levels at culture initiation) steadily during log phase growth, peaking with 4-fold increased expression at an OD_600_ of 0.9, followed by a steady decline during stationary phase growth (Figure 2A). Because the morphologic changes associated with the *Rv2190c* mutant suggested cell wall defects, which might in turn affect stress survival, we also analyzed expression of *Rv2190c* in during oxidative (cumene), disulfide (diamide) and detergent (SDS) stress conditions. As shown in Figure 2B, exposure of *M. tuberculosis* to 0.1% SDS resulted in a 4-fold increase in *Rv2190c* expression, indicating that Rv2190c may be involved in the maintenance of the *M. tuberculosis* cell wall.

To follow up on this transcriptional observation, we assessed the ability of the *Rv2190c* mutant to tolerate exposure to lysozyme, a cell wall-damaging enzyme. Compared to wild-type or complemented strains, the *Rv2190c* mutant exhibited a 30% reduction in cell viability (p<0.05) following a 24 hour treatment with 1 mg/ml lysozyme (Figure 2C). To examine if these cell wall-related phenotypes reflected altered lipid levels in the *M. tuberculosis* cell wall, we analyzed the composition of membrane lipids for the wild-type and *Rv2190c* mutant strains. As shown in Figure 2D, the *Rv2190c* mutant exhibited a displacement of PDIMs on the TLC plate when analyzed using hexane∶diethyl ether∶acetic acid (80∶20∶1 vol/vol/vol) solvent. To more clearly visualize this PDIM shift, we also utilized petroleum ether∶diethyl ether (90∶10 vol/vol) solvent, which can resolve DimA and DimB as two distinct species on the TLC plate. Use of this solvent revealed that, compared to wild-type *M. tuberculosis*, the *Rv2190c* mutant exhibited decreased DimA but increased DimB levels (Figure 2E). Taken together, these data indicate that Rv2190c plays a role in *M. tuberculosis* cell wall assembly during active bacterial growth and in response to cell wall disruption.

### The M. tuberculosis Rv2190c mutant is attenuated in vivo

**Figure 3:**
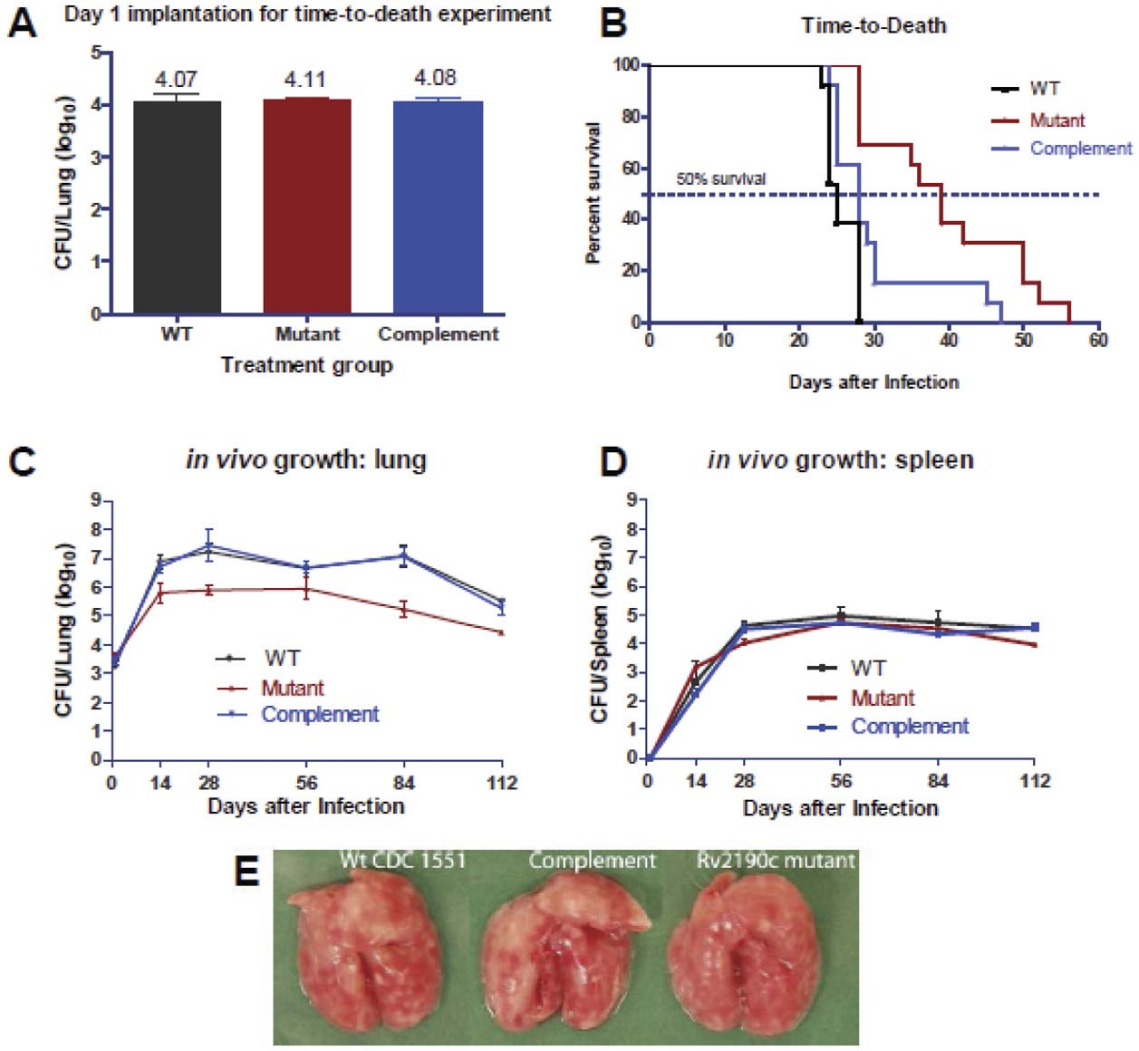
The *M. tuberculosis Rv2190c* mutant is attenuated *in vivo*. **A.** *M. tuberculosis* aerosol implantation in the lungs of BALB/c mice for the *Rv2190c* mutant, complement and WT (CDC1551) strains for the time-to-death experiment, determined day 1 post-infection. **B.** Survival curves for the time-to-death experiment. **C–D.** Lung (C) and spleen (D) CFU counts for BALB/c mice infected via aerosol with ∼3.4 log_10_CFUs of WT, *Rv2190c* mutant and complement *M. tuberculosis* strains. **E.** Gross pathology of infected mouse lungs at 112 days post-infection.

The alteration in PDIM of the *Rv2190c* mutant was strongly suggestive that this strain may be attenuated for growth and virulence *in vivo*. To test this hypothesis, we analyzed the time-to-death of BALB/c mice infected by aerosol with a high dose of either wild-type, *Rv2190c* mutant or complement strain of *M. tuberculosis*. Implantation, as determined by measuring lung CFUs the day after infection, indicated that the mice were equally infected with approximately 4.1 log_10_CFUs of the appropriate *M. tuberculosis* strain (Figure 3A). Despite receiving equivalent bacteria doses, the median survival for mice infected with the *Rv2190c* mutant (median: 39 days) was significantly longer than with mice infected with either the wild-type or complement strains (median: 25 and 28 days, respectively) (Figure 3B). Logrank comparison indicated that this difference between the median survival for the complemented strain and the mutant strain was statistically significant (28 versus 39 days, p = 0.0089); this difference could be due to the defective growth of the mutant strain that was observed *in vitro* (Figure 1A). These data suggested that lack of full-length Rv2190c renders *M. tuberculosis* less virulent in the mouse.

**Figure 4:**
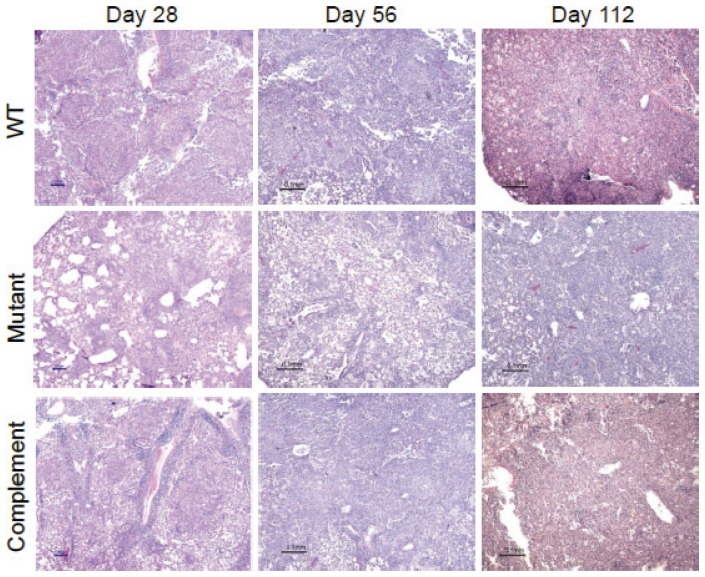
The *M. tuberculosis Rv2190c* causes decreased lung histopathology in the mouse. Formalin-fixed lung sections from mice infected with WT, *Rv2190c* mutant and complement strains were stained with hematoxylin and eosin and visualized using light microscopy. Scale bar = 0.1 mm.

To determine if the observed attenuation in the time-to-death study was associated with decreased bacterial burden in the mice, we again infected BALB/c mice by aerosol with wild-type, *Rv2190c* mutant and complement strains, and sacrificed 4 mice from each group for CFU determination at days 1, 14, 28, 56, 84 and 112 post-infection. In this model system, we found that the *Rv2190c* mutant was attenuated for growth in the mouse lungs, as 1–2 log_10_CFUs fewer were consistently recovered from mice infected with this strain across the time points, excluding day 1 (Figure 3C). In contrast, no difference in CFUs was detected in the spleen (Figure 3D), indicating that the *in vivo* growth attenuation of the *Rv2190c* mutant was specific to the mouse lungs. In addition, the lung gross pathology was noticeably reduced in mice infected with the *Rv2190c* mutant strain, with fewer caseating lesions when compared to the lungs from mice infected with wild-type or complement *M. tuberculosis* strains (Figure 3E). Lung histopathology analysis indicated reduced cellular infiltrate in the lungs of mice infected with the *Rv2190c* mutant strain compared to the lungs of mice infected with WT or complement strains (Figure 4). Taken together, these data indicate that lack of Rv2190c attenuates *M. tuberculosis* in both growth and virulence in the lungs in a murine model of TB.

### Rv2190c is expressed and immunogenic during mycobacterial infection

**Figure 5:**
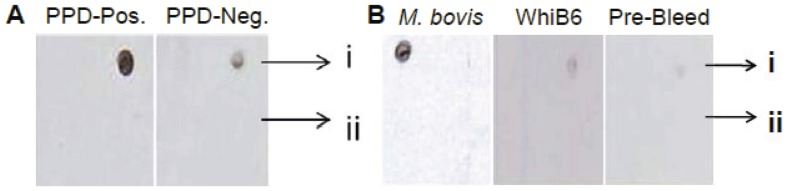
Immunogenic properties of the Rv2190c protein. N-terminal truncated Rv2190c was purified and analyzed for immunogenic properties by Dot-Blot ELISA against various sera. **A.** Immunogenic response in the human serum of an individual with latent TB (PPD-Pos.) (left panel) as opposed to a negligible response in a PPD negative human serum (right panel). **B.** Immunogenic response in serum from an *M. bovis* infected rabbit (left panel) and weak responses from sera raised against WhiB6 protein (middle panel) or pre-bleeds (right panel). i) 2ng of Rv2190c and ii) 2ng of BSA.

Our data indicated that Rv2190c influences the composition and structure of the *M. tuberculosis* cell wall, which raises the possibility that the Rv2190c protein might be located within the cell wall, possibly surface-exposed. Furthermore, Målen and colleagues have reported that this protein is exported out of the *M. tuberculosis* cytoplasm [13]. To address this possibility, we analyzed whether human sera from individuals with latent *M. tuberculosis* infection (*i.e.*, skin test [PPD] positive) could identify the Rv2190c protein. As shown in Figure 5A, serum from a PPD-positive individual reacted robustly with purified Rv2190c, compared to serum from a PPD-negative individual. In addition, serum from *M. bovis*-infected rabbits also showed a response to Rv2190c when tested by Dot-Blot ELISA (Figure 5B), while neither the serum raised against WhiB6 (non-specific control) protein or pre-bleed rabbit serum showed a reaction. Thus, these data indicate that Rv2190c is expressed, likely surface-exposed by *Mycobacteria* during infection, and, importantly, our data also demonstrate that this protein is immunogenic in *M. tuberculosis*-infected individuals.

## Discussion

The *M. tuberculosis* gene *Rv2190c* is described as encoding a hypothetical protein containing an NlpC/P60 domain. Here, we have characterized the role of this gene and its protein product, showing that Rv2190c is involved in cell wall maintenance and composition, is necessary for standard growth and virulence *in vivo*, and is expressed, possibly surface-exposed and immunogenic in the mammalian host. These findings provide significant groundwork towards understanding the role of this “hypothetical” protein, and, importantly, suggest that Rv2190c has potential translational utility that should be further investigated.

While our data demonstrate that *Rv2190c* is not essential, lack of this gene was associated with a growth defect *in vitro* (Figure 1A), in agreement with Sassetti's findings [21]. These findings are also consistent with a role for normal growth and development documented for NlpC/P60 domain-containing proteins in other bacteria, such as CwlF and LytF from *Bacillus subtilis* [6], [22], p75 from *Lactobacillus casei* B23 [23], p60 of *L. monocytogenes* [24], and cgR_1596 and cgR_2070 of *Corynebacterium glutamicum* [20]. However, in these bacteria the effect on growth was largely due to septational changes manifested by a lack of a functional hydrolase domain, and this was not the case with the *Rv2190c* mutant, as it did not show any septation defects (Figure 1C). This could be due to the fact that the transposon insertion in the mutant is C-terminal to the hydrolase domain containing the DCSG and GD motifs and the histidine residue. However, the growth defect implicated that hydrolysis to generate daughter cells may not be the sole function for this gene product. We have further demonstrated that this attenuated growth may be associated with the role of Rv2190c in cell wall integrity, as the *M. tuberculosis Rv2190c* mutant exhibited altered PDIMs (Figure 2D), increased susceptibility to lysozyme (Figure 2C), and altered gross morphology (Figure 1B) similar to the NlpC/P60-domain-containing proteins from *M. marinum*, *iipA* and *iipB* [4].

In addition to *in vitro* growth defects, the *Rv2190c* mutant also displayed growth alterations *in vivo* (Figure 3). The mutant showed enhanced survival in the mice in comparison to the wild-type and complemented strains and was attenuated for growth in the lungs (Figure 3B,C), also similar to the results obtained by Gao *et al*. for the *M. marinum* orthologues in zebrafish [4]. It is possible that the decreased bacterial burden of the mutant in the mouse lungs was due to a general growth defect as was observed *in vitro* (Figure 1A); however, the mutant was not attenuated for growth in the mouse spleen, in terms of either colony forming units (Figure 3D) or gross morphology (data not shown). This lack of attenuation in the spleen suggests that the phenotype observed in the lungs may not be due to generally defective growth. This was consistent with the findings of Cox *et al.*, who showed that PDIMS determined tissue specific replication of *M. tuberculosis* in mice [25]. The specific mutants tested (*pps*, *fadD28* and *mmpL7*: genes that are involved in either PDIM synthesis or export) were altered for PDIM levels, showed alterations in colony surface morphology and were attenuated for growth in mouse lungs but not spleen, similar to our data.

Although the transposon insertion in our *Rv2190c* mutant was downstream of the encoded NlpC/P60 domain, the function of the Rv2190c protein was altered, resulting in altered cell morphology and PDIMs. This phenotype could be due to the protein misfolding due to the insertion sequence and/or could be due to the inability of the interrupted protein to properly interact with binding partners or substrates. The complement strain was not able to restore growth fully to the wild-type level; however, gross colony morphology, lysozyme resistance, and *in vivo* phenotypes of the complemented strain suggest that this Rv2190c was functionally restored in our complemented strain. *Rv2190c* is not predicted to be part of an operon [26], [27], and the functional complementation shown in most of our assays suggest that the phenotypes associated with the *Rv2190c* mutant are not due to polar effects. It is possible that the intermediate *in vitro* growth of the complemented strain, as well as the intermediate total time-to-death during infection of mice with this strain, could be due the presence of the mutated/misfolded Rv2190c mildly interfering with the full-length protein.

Rv2190c has been shown to be an exported protein. It has a functional signal sequence in the N-terminal and has been shown to be secreted into the cell culture filtrate of *M. tuberculosis* [8], [13], another commonality with quite a few of the NlpC/P60 domain containing proteins that have been shown to be secreted and immunogenic. Taken together, the data from this study and others indicate that the N-terminus likely serves as signal sequence to export the protein to the cell surface to be either anchored and/or secreted (similar to p40 and p75 of Lactobacilli), and the hydrolase domain and C-terminus engage substrates and other proteins [8]. As *M. tuberculosis* has five NlpC/P60 genes and five Rpfs [28], it is tempting to speculate that specific associations exist between the two, enabling functional interactions. However, unlike the Rpfs, the genes encoding the NlpC/P60 family proteins are not redundant as both *Rv1477* and *Rv1478* mutants in *M. marinum* and *Rv2190c* mutant in *M. tuberculosis* show phenotypic differences. It is possible that each protein interacts with different molecules thereby decreasing the chances of redundancy.

We have demonstrated for the first time that the hypothetical protein Rv2190c is important for *M. tuberculosis* growth and virulence. While not an essential protein, lack of Rv2190c weakens the bacterial cell wall, indicating that inhibition of Rv2190c may allow for better penetration of other anti-mycobacterial compounds. Thus, our results suggest that Rv2190c should be further investigated as a possible drug target. In addition, our data showing that Rv2190c is expressed and immunogenic in the human host indicate that this protein could also be further studied for translational development.
